# Characterization of LysBM1, a novel high-penetrating phage lysin targeting enterohemorrhagic *Escherichia coli*

**DOI:** 10.3389/fvets.2025.1631293

**Published:** 2025-07-11

**Authors:** Haitao Liu, Lichang Sun, Quan Sun, Shiqiang Zhang, Ran Wang

**Affiliations:** ^1^College of Veterinary Medicine, Shaanxi Stem Cell Engineering Research Center, Northwest A&F University, Yangling, China; ^2^Key Laboratory of Livestock Biology, Northwest A&F University, Yangling, China; ^3^Key Laboratory of Food Quality and Safety of Jiangsu Province-State Key Laboratory Breeding Base, Institute of Food Safety and Nutrition, Jiangsu Academy of Agricultural Sciences, Nanjing, China

**Keywords:** Enterohemorrhagic *Escherichia coli*, phage, lysin, penetrating, food safety

## Abstract

We investigated the lytic activity of the bacteriophage endolysin (lysin) LysBM1, which has a predicted molecular mass of ~25 kDa and is encoded by an enterohemorrhagic *Escherichia coli* (EHEC) phage. LysBM1 was over expressed in *Pichia pastoris* GS115 and purified protein exhibited optimal bactericidal activity *in vitro*. Herein, the antimicrobial and penetrating activity of LysBM1 were studied. Our results showed that the lysin displayed a broad lytic spectrum and high activity, with *in vitro* treatment killing all 21 of the clinical EHEC strains tested. Laser confocal microscopy showed Alex Fluor 610-X conjugate-labelled LysBM1 inside EHEC E5 cells, while confocal laser scanning microscopy revealed that cells exposed to LysBM1 for 5 min suffered deformation and disruption of the cell wall. A LysBM1 protein containing mutations within the active sites (LysBM1-M) retained bactericidal activity despite its inability to hydrolyze peptidoglycan. Both LysBM1 and LysBM1-M could penetrate the *E. coli* outer membrane, confirming that LysBM1 had membrane-disrupting activity. The present results suggest that LysBM1 has the potential to be used as an alternative therapeutic agent against pathogenic EHEC strains.

## Introduction

Enterohemorrhagic *Escherichia coli* (EHEC) make up a subset of the Shiga toxin-producing *E. coli*. They are important zoonotic pathogens, causing diarrhea, hemorrhagic enteritis, and even death in animals. Antibiotic abuse has resulted in the emergence of antibiotic-resistant EHEC strains, including multidrug-resistant “superbugs,” which are extremely harmful to both human health and the livestock industry. Therefore, there is an urgent need for effective prevention and control strategies for EHEC.

Bacteriophage lysins are highly effective “enzymatic antibiotics.” The increasing prevalence of antibiotic resistance has led to significant global interest in the application of lysins in animal disease prevention and control. Lysins are derived from bacteriophage; however, bacteriophage has been limited because of their narrow lysis spectrum and the fact they are mutable. Bacteriophage lysins are lysozymes that break down the bacterial cell wall. They are encoded by the phage genome and help in the release of viral particles at the terminal stage of phage reproduction (1). Typical lysins have two independent functional domains: the N-terminal catalytic domain (CD) and the C-terminal cell wall-binding domain (CBD), which are responsible for hydrolytic activity and cell wall-binding activity, respectively (2). Generally, the N-terminal CD specifically breaks down chemical bonds within peptidoglycans. Lysins can be divided into several classes according to the chemical bonds that they target, including lysozyme, amidase, and endopeptidase. In general, lysins targeting Gram-negative bacteria only contain a CD, with no CBD, and range in size from 12–20 kDa. Lysins are genus/species-specific and highly effective at digesting peptidoglycan, allowing them to break down bacterial cell walls, including those of multidrug-resistant Gram-positive bacteria, with high efficiency (3). Many studies have documented the application of lysins in animal disease prevention and control, production of bioenzyme antibiotics, and food preservation (4, 5). For example, lysins (6, 7) have been used to, treat dairy cow mastitis (8), *Streptococcus equi* infection (9), canine pyoderma (10), and *E. coli* infection in swine (11). In addition, lysins can effectively clear infections caused by *E. coli* strains (12, 13).

In the present study, we identified lysin LysBM1 by genome sequencing (GenBank accession number: MH607138). The lysin had a predicted molecular mass of ~25 kDa and is encoded by EHEC phage V_EcoM_BM1. Histidine-tagged LysBM1 was overexpressed in *Pichia pastoris* GS115 and purified by NI-NTA chromatography. The purified protein exhibited optimal bactericidal activity *in vitro*. The MIC of LysBM1 against EHEC strain E5 was 86 ± 6 *μ*g/mL, and it demonstrated a broad spectrum of activity against all EHEC strains tested. We also carried out analysis of LysBM1 cell wall-penetrating activity. Based on these results, we conclude that LysBM1 represents a promising therapeutic agent against EHEC.

## Materials and methods

**Figure 1:**
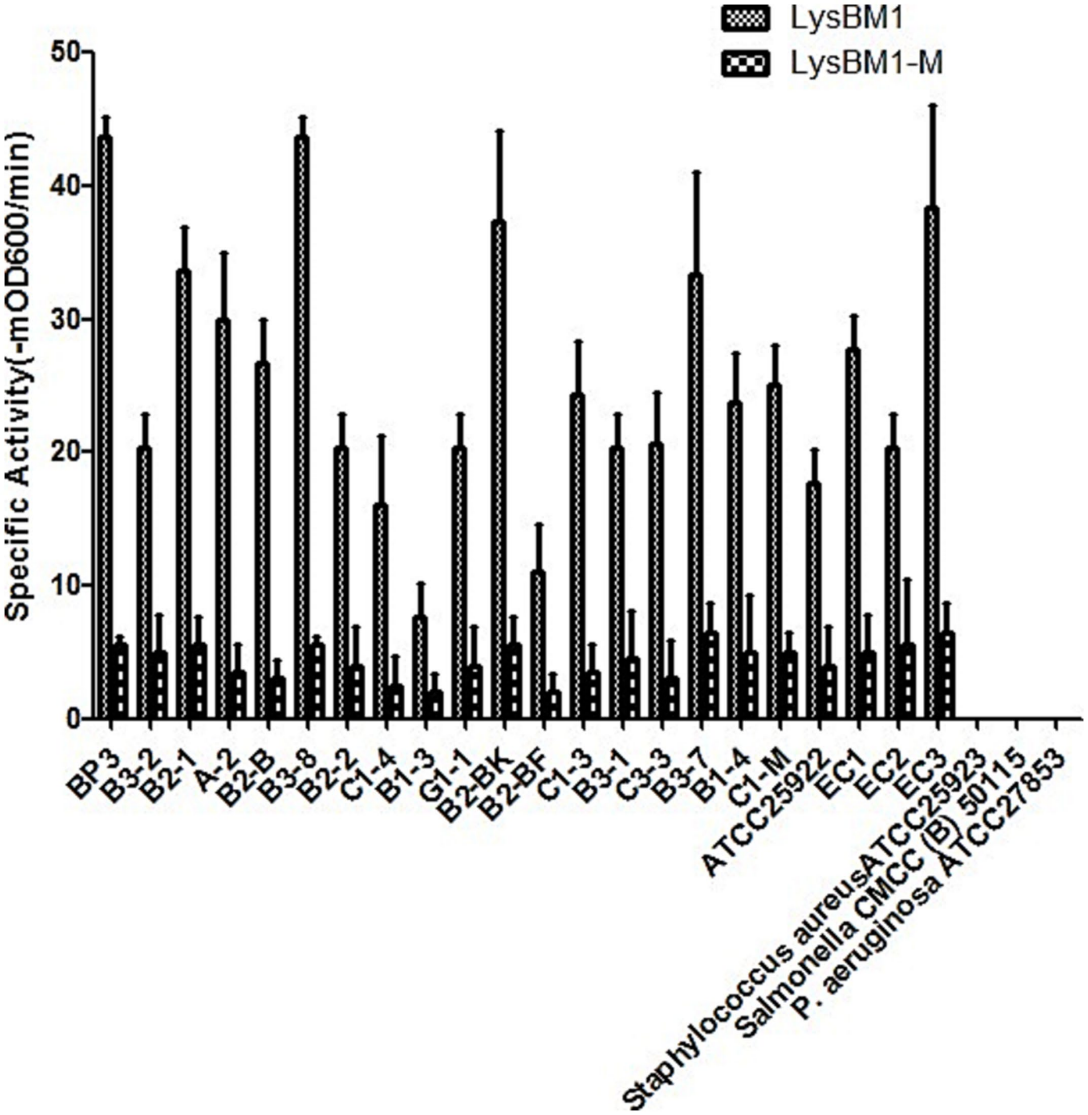
Lytic activity of LysBM1 (final concentration, 100 μg/mL) against different *Escherichia coli* strains *in vitro*. Lytic activity was defined as the reduction.

Bacterial strains and culture conditions. *E.coli* strain ATCC 25922, *Staphylococcus aureus* ATC25923, sa*lmonella* CMCC (B) 50115, P*.aeruglnosa* ATCC27853 were stored in our laboratory; a collection of 20 clinical EHEC strains isolated from milk samples from cows with bovine mastitis. All bacterial strains used in this study are described in Figure 1. *E. coli*, *Pseudomonas aeruginosa*, and *Salmonella enterica* strains were cultured in Luria-Bertani (LB) broth at 37°C. *Staphylococcus aureus* was cultured at 37°C in tryptic soy broth. *P. pastoris* GS115, used as a host for protein over-expression, was grown at 30°C in YPD medium.

Construction of expression plasmids and purification of recombinant proteins. The DALI online software was used to compare and analyze the structure of LysBM1 with the structure in the database; The ConSurf server can be used to predict protein conserved sites and active sites. The ConSurf server (http://consurftest.tau.ac.il/) was used to analysis of the structure and sequence of LysBM1. We foud that the peptidoglycan hydrolysis active sites of LysBM1 were located at N40 and R53 (LysBM1 GenBank accession number: MH607138, URL: https://www.ncbi.nlm.nih.gov/nuccore/MH607138.1/). In order to detect LysBM1 penetrating activity, we designed a LysBM1 peptidoglycan hydrolytic activity inactivating mutant LysBM1-M which contains mutations N40G and R53G. LysBM1-M were synthesized by Genscript (Nanjing, China) and cloned into the pET-32a expression vector (Novagen, Madison, WI) using *NdeI* and *XhoI* restriction sites. The resulting plasmids, expressing the target proteins fused to a 6His-tag at the C-terminus, were transformed into electrocompetent *E. coli* BL21 (DE3). Isopropyl *β*-D-1-thiogalactopyranoside-mediated induction of protein expression led to growth arrest of logarithmic *E. coli* BL21 cells. Therefore, the LysBM1-6His and LysBM1-M-6His construct were sub-cloned into *P. pastoris* GS115 expression plasmid pPIC9K using restriction enzymes EcoRI and NotI, resulting in recombinant plasmids pPIC9K-LysBM1-6His and pPIC9K-LysBM1-M-6His. The ligation mixtures were electroporated into *E. coli* Top10 cells and transformants were selected on plates containing 10 *μ*g/mL ampicillin. The presence of the correct insert was verified by commercial DNA sequencing (Genscript, Nanjing, China). pPIC9K-LysBM1-6His and pPIC9K-LysBM1-M-6His (8 *μ*g) were linearized using SalI and transformed into *P. pastoris* GS115 by electroporation at 1.5 kV, 25 *μ*F, and 200 *Ω* for 5 msec. Transformants were selected on MD plates before His+ transformants were selected on YPD plates containing 5 mg/mL Geneticin (G418). PCR was used to verify positive transformants identified by G418 selection. The positive colonies were then inoculated into 100 mL of BMGY medium and cultured at 30°C with constant shaking at 250 rpm until reaching an optical density at 600 nm (OD_600_) of 3.0. Cultures were then centrifuged at 4000 *g* for 10 min and the cell pellets were resuspended in 20 mL of BMMY medium before being continuously induced for 96 h at 30°C with shaking at 250 rpm. Methanol was maintained at a concentration of 1% (v/v). Sodium dodecyl-sulfate polyacrylamide gel electrophoresis (SDS-PAGE) was performed to confirm the expression of LysBM1. The supernatant was separated from the culture, and the purification was completed using a Sepharose CL-6B column (GE Healthcare, Somerset, NJ). LysBM1 and LysBM1-M were then lyophilized, diluted in phosphate-buffered saline (PBS) using pyrogen-free reagents, aliquoted, and stored at −20°C. The protein concentration was determined using a bicinchoninic acid Protein Assay Kit (Beyotime, Shanghai, China).

Quantification of LysBM1 activity. Enzyme activity was quantified by turbidimetric reduction assay in a spectrophotometer. Briefly, EHEC strain E5 was grown to a final OD_600_ of 0.8 (10^8^ CFU/mL). One unit of LysBM1 activity was defined as the highest dilution that decreased the absorbance by 50% within 15 min at 37°C, as previously described (14–16). The MIC assay was carried out as described previously (17) with some modifications. All MIC assays were performed in LB broth. After 1 U of LysBM1 was mixed with the suspension of *E. coli* cells, the decrease in viable cells corresponding to the loss of turbidity was also tested by plating aliquots from the lytic assay at various time points (5, 15, 30, and 60 min) onto LB agar to obtain CFU counts. The lytic activity of LysBM1 at different pH values was measured using a universal buffer as previously described (18). The buffer was prepared by mixing equal parts 20 mM boric acid and 20 mM phosphoric acid, followed by titration with sodium hydroxide from pH 3–12. The lytic activity of LysBM1 was tested in the buffer at different pH levels and at temperatures ranging from 5–55°C. All of the experiments described above were performed in triplicate, and bacterial cells treated with LB broth were used as blank controls.

Laser confocal microscopy analysis. An aliquot of EHEC E5 cells (1 × 10^7^ CFU/mL) was co-incubated with lysozyme (Sangon Biotech, Shanghai, China) or LysBM1 labeled with WGA 610-X conjugate (Invitrogen, Carlsbad, CA) for 30 min. After washing three times with PBS, the E5 cells were observed by laser confocal microscopy.

Confocal laser scanning microscopy analysis. Mid-logarithmic phase EHEC E5 cells (10^9^ CFU/mL) were incubated with LysBM1 at 1 × MIC for 10 and 30 min. Propidium iodide (final concentration 50 μg/mL; P4170, Sigma Aldrich, Steinheim, Germany) was then added to each culture and the cells were incubated for a further 30 min at 4°C in the dark before being washed twice and re-suspended in 10 mM PBS (pH 7.2). Cell suspensions were transferred into glass-bottom dishes for imaging. Untreated cells (no LysBM1) were used as the control. Cell images were then obtained using an LSM 710 confocal laser scanning microscope (Carl Zeiss, Oberkochen, Germany).

Determination of membrane permeabilization. Mid-logarithmic phase EHEC E5 cells (10^9^ CFU/mL) were incubated with LysBM1 or LysBM1-M at 1 × MIC for 30 min. A 1.5-mM stock solution of propidium iodide in distilled water was diluted with phosphate buffer to a concentration of 30 mM and stored in the dark at 4°C. A 500-μl volume of treated cell suspension was mixed with 500 μL of the dye solution and incubated at room temperature for 15 min in the dark. Samples not treated with the lysin and phosphate buffer were used as the controls. The background signal was determined in the absence of propidium iodide, with fluorescence being measured using a spectrofluorometer (LS-55, Perkin Elmer, Waltham, MA) at excitation and emission wavelengths of 485 and 635 nm, respectively. The slit width was 5 nm. The results were expressed as propidium iodide uptake factors, which were calculated as described previously (19).

Statistical Analysis Data organization was completed using Microsoft Excel 2021 (Microsoft Corporation, Redmond, WA, USA), and graphs were generated using Origin 2022 (Origin Lab Corporation, Northampton, MA, USA). The data are expressed as means ± SD. Statistical significance was determined based on one-way analysis of variance (ANOVA) in appropriate conditions using GraphPad Prism 8 software. Significance was determined at *p* < 0.05.

## Results

**Figure 2:**
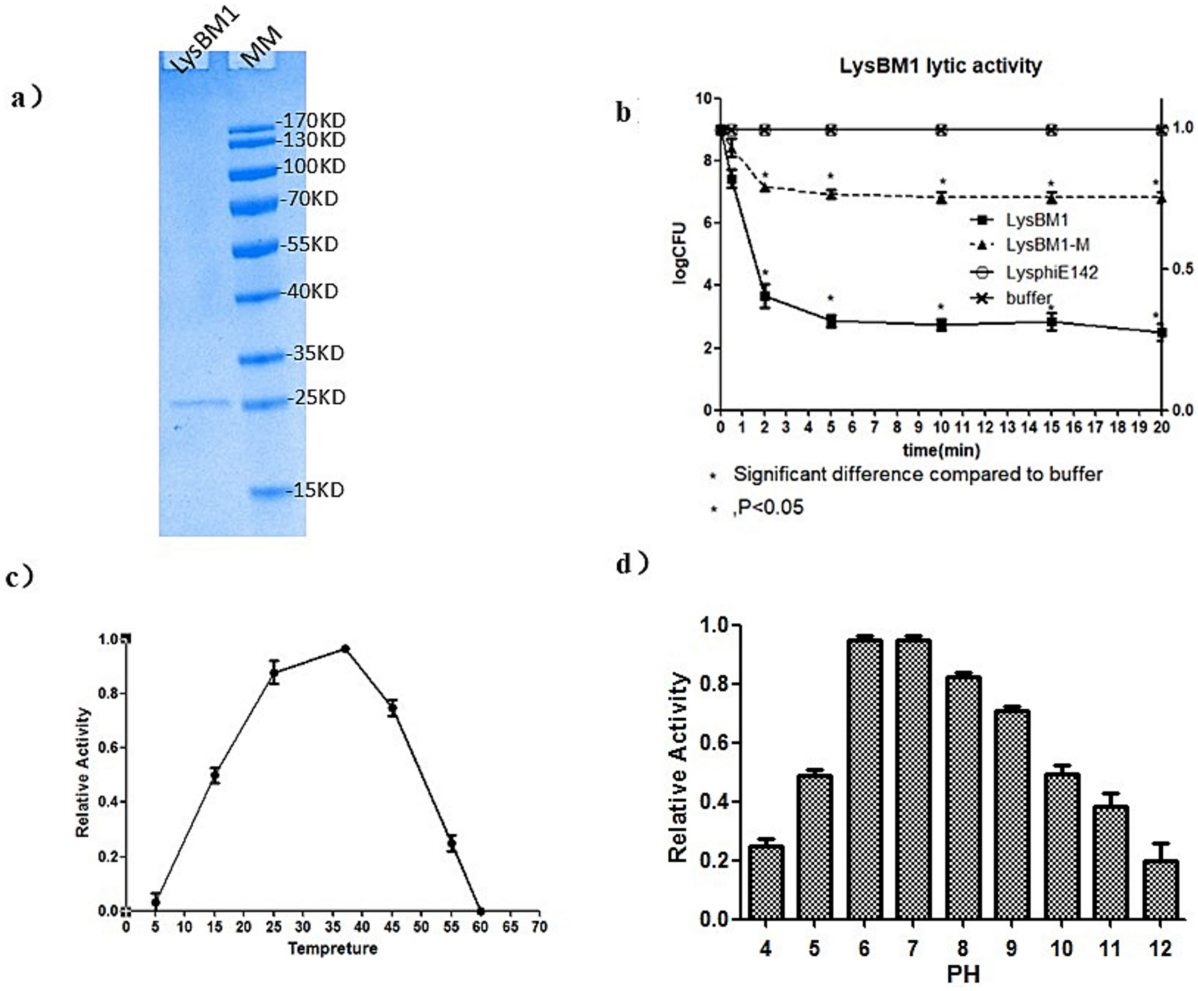
Characteristics of LysBM1 activity. **(a)** SDS-PAGE (12%) analysis of purified LysBM1 (predicted MW = 15 kDa; loading concentration: 0.1 mg/mL). M, protein molecular mass markers. **(b)** Lytic activity against *Escherichia coli* E5 *in vitro*. Decreases in CFU were monitored following the addition of LysBM1. LB buffer was used as a control. Changes in cell numbers were recorded as log CFU/ml and were determined by serial dilution and plating onto LB agar plates. **(c)** Relative activity of LysBM1 against *Escherichia coli* E5 cells in buffer incubated at different temperatures. **(d)** Relative activity of LysBM1 against *Escherichia coli* E5 cells in buffer adjusted to different pH levels in turbidity within 15 min (−mOD_600_/min). Error bars show the standard errors of three independent assays.

Characteristics of LysBM1. The purified LysBM1 was shown to be highly pure (N93%) by 12% SDS-PAGE analysis (Figure 2a) and had a MIC of 86 ± 6 *μ*g/mL against EHEC strain E5. Following the addition of LysBM1, the CFU of the EHEC strain E5 cell suspension decreased rapidly (within 5 min), while the CFU of the untreated cell suspension continued to increase (Figure 2b). This decrease in cell density of the LysBM1-treated culture corresponded to a 5–6-log_10_ reduction in CFU (Figure 2b). As expected, LysBM1 elution buffer alone had no effect. We then examined the influence of temperature and pH on the activity of LysBM1 and found that the purified protein demonstrated high-level lytic activity at temperatures ranging from 20–45°C (Figure 2c). LysBM1 also retained a high level of activity against EHEC strain E5 cells across a broad pH range (pH 3.5–9.5), with maximum activity observed at pH 7.2 (Figure 2d).

LysBM1 lyses live *E. coli* in a turbidity reduction assay. As shown in Figure 1, LysBM1 demonstrated effective lytic activity against all tested EHEC strains, but LysBM1 did not appear to be active against any of the other species tested (Figure 1). The observed variability of LysBM1 activity against the tested strains may be associated with the resistance type and the structure of the cell wall. LysBM1 had broad-spectrum activity against all EHEC strains but not against the other species tested. EHEC BP3 strain is the most sensitive to LysBM1. In short, LysBM1 represents a promising therapeutic agent against EHEC.

**Figure 3:**
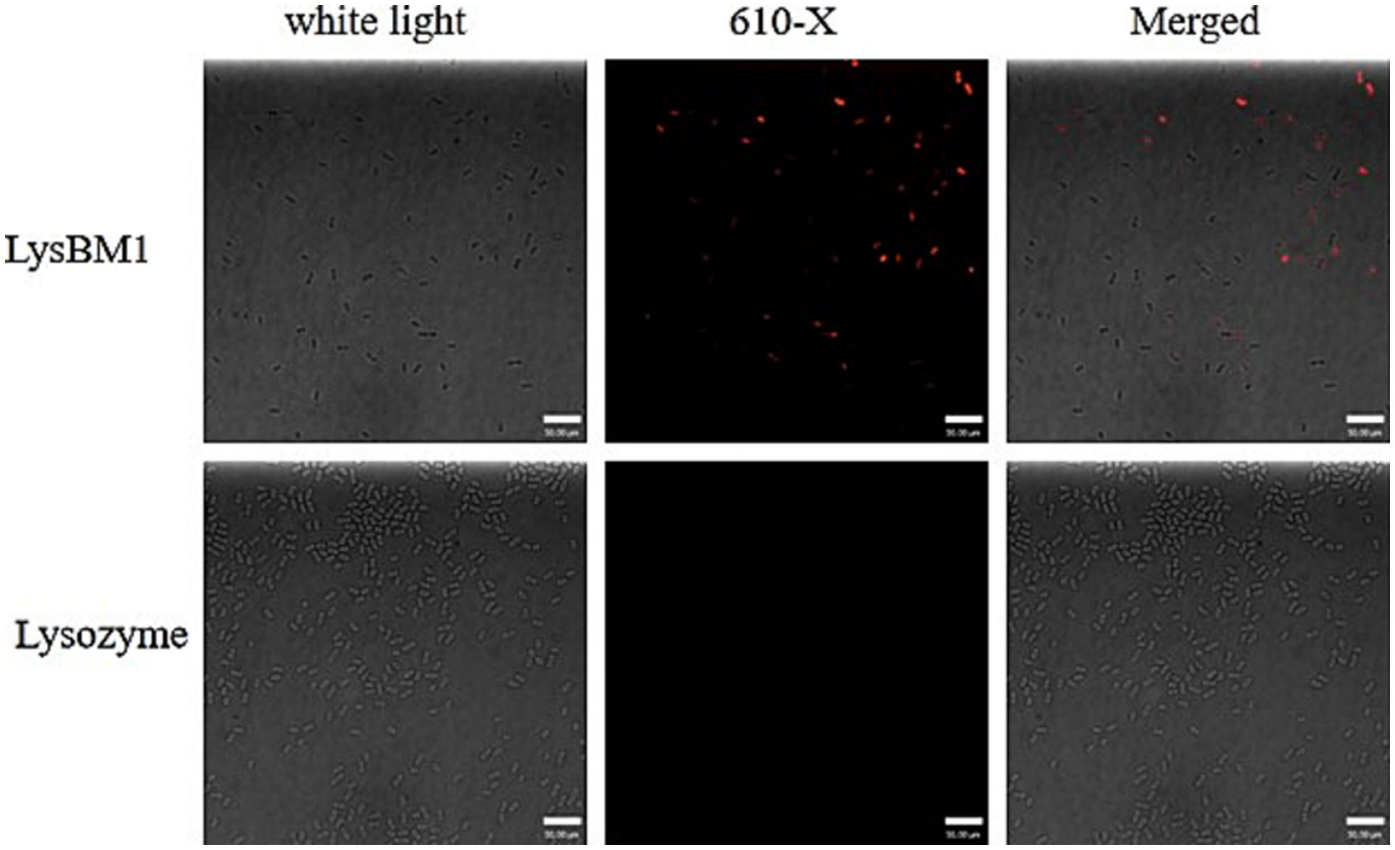
Ability of LysBM1 to permeate into *Escherichia coli* cells. *Escherichia coli* cells were co-incubated with Lysozyme or LysBM1 labeled with WGA 610-X conjugate (Invitrogen). The labeled LysBM1 was observed in *Escherichia coli* E5 cells by laser confocal microscopy but was not found in Lysozyme control cells.

Laser confocal microscopy analysis. We next investigated whether LysBM1 could enhance the efficiency of host-cell permeation. To determine whether LysBM1 could permeate host cells, EHEC E5 cells were co-incubated with lysozyme or LysBM1 labeled with WGA 610-X conjugate. The labeled LysBM1 was subsequently observed inside the EHEC E5 cells by laser confocal microscopy but was not observed in lysozyme-treated control cells (Figure 3). As shown in Figure 3, LysBM1 treatment resulted in more efficient and specific permeation of the host cells compared with lysozyme treatment, despite the two enzymes having similar structures.

**Figure 4:**
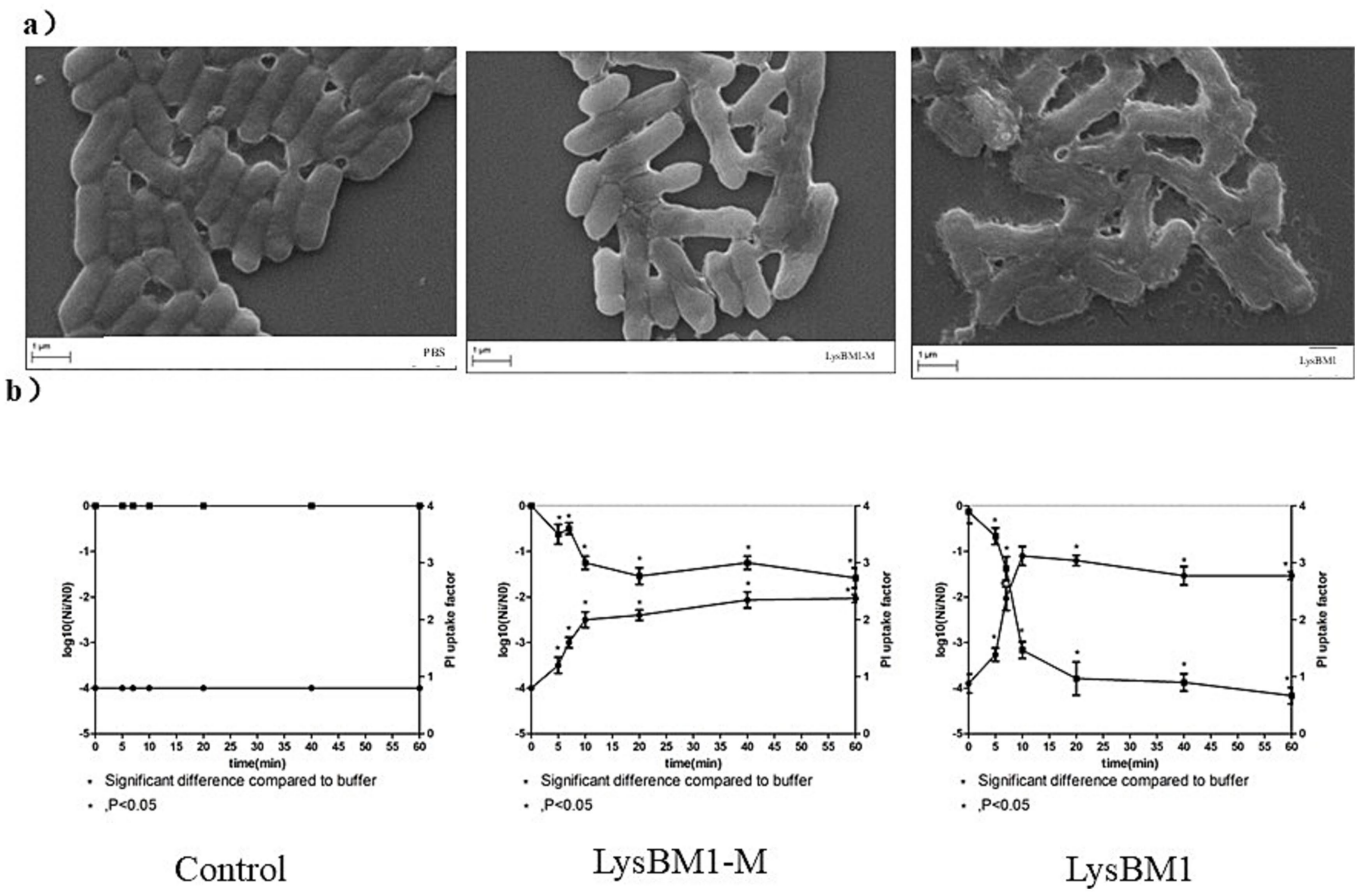
Characteristics of LysBM1 activity. **(a)** Confocal laser scanning microscopy images of *Escherichia coli* E5 cells exposed to LysBM1 or LysBM1-M (final concentration, 100 μg/mL) or PBS. LysBM1 caused deformation of the cell wall surface, while cells exposed to LysBM1-M, which is unable to hydrolyze peptidoglycan, demonstrated a localized weakening of the cell membrane. Bar sizes, 1 μm. Magnification, 10,000×. **(b)** Determination of the surviving fraction and the degree of membrane permeabilization of *Escherichia coli* E5 cells, as determined by the propidium iodide uptake factor, following treatment with LysBM1 or LysBM1-M (final concentration, 100 μg/mL) or PBS. Mean values ± standard deviation are shown.

Determination of membrane permeabilization by confocal laser scanning microscopy and PI uptake assay. We foud that LysBM1-M (containing mutations N40G and R53G) had no peptidoglycan hydrolysis activity (data not shown). Confocal laser scanning microscopy analysis showed that cells exposed to LysBM1 for 5 min suffered deformation and disruption of the cell wall, resulting in cell lysis (Figure 4a). Similar disruption was not observed in the control group. Cells exposed to LysBM1 demonstrated a localized weakening of the cell wall, resulting in extrusion and rupture of the cell membrane. However, cells exposed to LysBM1-M, which is unable to hydrolyze peptidoglycan, also demonstrated a localized weakening of the cell membrane (Figure 4a).

The number of *E. coli* cells surviving after PBS or LysBM1-M/LysBM1 treatment and the degree of membrane permeabilization (measured by the uptake of propidium iodide) were determined, and the results, presented as a function of the exposure time, are shown in Figure 4b. Very little inactivation was observed during the first stage of treatment (i.e., shoulder period), while in the second stage, the number of viable cells decreased exponentially. After 20 min, the inactivation rate leveled off and remained constant over time (i.e., the tailing period). Overall, subjecting *E. coli* E5 to LysBM1 treatment for more than 20 min reduced the number of viable cells by 3.0 log units. When E5 cells were treated with LysBM1-M, the shoulder period disappeared (or was at least not apparent from the sampling points examined in this study). However, the majority of cells were inactivated within 5 min, and exposure to LysBM1-M for more than 20 min reduced the number of viable *E. coli* E5 cells by 1.5 log units. These results indicated that even though LysBM1-M did not have peptidoglycan hydrolase activity, it continued to be bactericidal, perhaps suggesting that LysBM1-M penetration itself was bactericidal.

The degree of permeabilization of *E. coli* cells, as measured by the uptake of propidium iodide, was time-dependent and could be divided into two or three stages, depending on the processing conditions (Figure 4b). In general, the onset of these stages corresponded well with the onset of the phases observed in the survival curves. The assays showed that both LysBM1 and LysBM1-M treatment resulted in an increase in the uptake of propidium iodide, with the uptake factors of LysBM1 and LysBM1-M increasing by 2 and 1, respectively. This result supported the conclusion that both LysBM1 and LysBM1-M could penetrate the *E. coli* outer membrane, and that LysBM1 had membrane-disrupting activity.

## Discussion

EHEC are a serious threat to public health and food safety. Phage-encoded lysins can effectively and specifically kill bacteria but cannot penetrate the outer membrane of Gram-negative species. Here, we identified lysin LysBM1, which has a predicted molecular mass of ~25 kDa and is encoded by an EHEC phage. We then showed that LysBM1 has strong cell-penetrating and lysozyme activities.

Lysins from Gram-positive bacterial phage have excellent bactericidal activity; however, as peptidoglycan is not exposed on the surface of the Gram-negative outer membrane, the lytic activity of these lysins against Gram-negative bacteria is limited. The Gram-negative bacterial cell wall consists of the outer membrane and the periplasmic space. The cell wall contains a thick lipid layer in addition to the peptidoglycan and is considered to be a permeable barrier. However, it prevents the entry of hydrophobic antibiotics, reagents, and proteins. Because of this more complex structure, lysins cannot directly bind to the peptidoglycan from the outside and are therefore unable to lyse cells without help (20, 21). Gram-negative phage lysins need to cross the outer membrane to hydrolyze the peptidoglycan and kill bacteria. Therefore, the effects of lysins on the bacterial outer membrane need to be enhanced without affecting activity.

To date, three different strategies have been attempted to increase the activity of Gram-negative lysins. The first uses physical or chemical methods to help the lysins cross the outer membrane, as evidenced by chloroform (22) or hydrostatic pressure (23) treatments allowing lysis of *P. aeruginosa* by lysin KZ144. In the second strategy, lysins are fused with cationic peptides or permeation domains (24, 25). Briers et al. (26, 27) designed antibacterial peptides and fused them with a lysin. The resultant recombinant enzyme effectively ruptured multidrug-resistant *P. aeruginosa*. The study found that net charge and hydrophobicity had prominent effects on lysin activity. Lukacik et al. (20, 21) fused T4 phage lysin with pesticin from a *Pasteurella pestis* phage and effectively ruptured *P. pestis* and pathogenic *E. coli* cells. Further, Liancheng et al. fused the C-terminus of *E. coli* phage lysin Lysep3 with permeation domain D8 (28). The resultant protein could directly kill *E. coli* cells *in vitro*. In the third approach, acidic amino acids are substituted with basic amino acids to modify the lysin charge. When non-functional acidic amino acids are substituted with the basic amino acid arginine, the net positive surface charge increases. As phospholipid bilayers carry abundant negative charges, the binding affinity between the lysin and the phospholipid bilayer should therefore increase (29, 30). Using this method, Roberto et al. modified the pneumococcal lysin so that it would lyse certain Gram-negative bacteria (31).

In summary, the major issue relating to Gram-negative bacterial lysins is the need to cross the bacterial outer membrane. Previous investigations have made significant headway in developing methods to overcome the barrier of the outer membrane, including modification of the physical/chemical conditions and fusion with cationic peptides or functional domains with membrane-permeation activity (32, 33). Recent studies have also identified Gram-negative lysins with *in vitro* activity; however, the in-depth mechanisms were not discussed. We isolated lytic phage V_EcoM_BM1 from a sewage EHEC isolate and used whole genome sequencing to identify the gene coding for lysin LysBM1. BLAST analysis showed that LysBM1 showed the greatest identity to the genome of *E. coli* O157 phage phiE142 (34), but the similarity was only 62%. Therefore, LysBM1 was considered to be a novel *E. coli* lysin. Protein homology modelling showed that LysBM1 and phiE142 were highly similar in their tertiary structures, as both contained a typical lysozyme-like structural domain. Recombinant LysBM1 expressed in *P. pastoris* was antibacterial and highly effective in penetrating the EHEC outer membrane. Electron microscopy and confocal laser scanning microscopy analyses clearly showed that LysBM1 crossed the bacterial outer membrane, suggesting that is has membrane-penetrating activity. Therefore, the present study expands our understanding of the way in which lysins affect the bacterial outer membrane and provides new ideas and a theoretical basis for designing highly efficient lysins, which will promote the widespread application of lysins as alternative therapeutic agents.

## Data Availability

The datasets presented in this study can be found in online repositories. The names of the repository/repositories and accession number (s) can be found at https://www.ncbi.nlm.nih.gov/nuccore/MH607138.
